# Gelsemium Sempervirens, Yellow Gels

**Published:** 1877-05-20

**Authors:** I. J. M. Goss

**Affiliations:** Marietta, Ga.


### GELSEMINUM SEMPERVIRENS, YELLOW GELS.

(WILD WOODBINE.)

[Extract from the Manuscript of Materia Medica (by I. J. M. Goss, A. M., M. D., Marietta, Ga.,) to be published by Subscription, and now in press at St. Louis, Mo.

BOTANICAL DESCRIPTION.—Gelseminum is the Bignonia Sempervirens of Linnæus, and the Gelsiminum Nitridum of Michaux and Purch. It is an evergreen, having twining, smooth, glabrous stem, with opposite, perennial, lanceolate, entire leaves, which are dark green above, but pale beneath, standing on short petioles. The flowers are yellow, and of an agreeable odor, and stand on axillary peduncles; the calix is very small, with five sepals; the corolla is funnel-shaped, with a spreading border, and with five lobes, nearly equal; stamens five; pistils, two; capsules, two-celled, compressed, flat, two partible; seeds flat, and attached to the margin of the valves. The berries are black. It is a beautiful evergreen, climbing plants and trees to the height of fifty to one hundred feet. It has beautiful yellow flowers in spring and is often cultivated for its shade, and as an ornament. The root is the part used in medicine. It is several feet in length, with scattered fibres of various sizes. The medical virtues reside in the bark of the root.

TOXICAL EFFECTS.— In over-doses Gelseminum will produce great relaxation and prostration, and finally paralysis of all the voluntary muscles; and pushed still further, it will paralyze the involuntary muscles, and thereby produce death, which it has done frequently.

It first affects the muscles of the eye producing also dimness of sight; then affects the sphincter muscles; then the respiratory, and finally the heart. In this stage the functions of the brain are not always abolished, but can be aroused by electricity and physical agitation of the body. In some cases, however, a deep stupor occurs, lasting until death or convalescence takes place. In some cases, when large doses have been taken, the brain becomes suddenly congested (from atony of the venous capillaries,) and a kind of apoplexy takes place. In this state life may often be saved by stimulants and galvanism freely applied to the spine.

THE SPHERE OF ACTION.—Gelseminum principally acts upon the motor side of the spinal cord, the brain and mucous membranes. The sensory nerves are not as profoundly affected by Gelseminum as by aconite. It will be seen at once that Gelseminum is a remedy for convulsions—first of the voluntary, then of the involuntary muscles. In chorea, hysteria, tetanus, puerperal spasms, and the spasms of all the hollow organs and also of the sphincter muscles Gelseminum will act promptly.

It is a valuable remedy in the hot stage of fevers of an active form. I had recently a case of remittent fever with dysentery, in a lady that was pregnant, and fearing to use quinia, I gave her Gelseminum in small, repeated doses, so as to keep up its effects, which checked the fever and aided very much in the treatment of the dysentery. In many local inflammations, especially where the mucous membranes are involved, I have found it a very valuable remedy, as in catarrhal affections. In hyperæsthesia and neuralgia it often gives relief promptly. In epidemic influenza, at any season of the year, where the attacks are attended with chilly sensations, a torpid, heavy sensation, with great aching of the back an limbs, this will be found to be the remedy in the disease.

It is the remedy in febrile or inflammatory conditions, where the pulse is large, full and quick, but soft, and the febrile manifestations are remittent; but aconite in those forms of fever or inflammation, attended with a small, hard, wiry quick pulse; and veratrum in those cases attended with a hard, full, bounding incompressible pulse. For want of a knowledge of these facts, many practitioners meet with sad disappointment in the use of arterial sedatives, and become discouraged in their use. I was called in consultation with a physician, a short time since, to see a child that was laboring under an attack of broncho-pneumonia. The fever was high, with deep congestion of the lungs, with great dyspnœa; pulse quick, wiry, hard, small, evidently showing a want of power in the heart to propel the blood in volume; hence the increased rapidity of the heart's strokes. He at once proposed veratrum, but I preferred aconite in small doses; but in spite of all my arguments, he would give veratrum, and that in large doses—one drop every hour, to a child of some four or five years of age, of feeble stamina.

The result was, that after two or three doses the child began to sink. The veratrum produced violent vomiting, cold, clammy sweat, and prostration, from which it could not be aroused, but died in a short time.

In infantile remittents, when due to, or connected with the irritability consequent upon teething, worms, or intestinal irritation, or when caused by malarial influences, Gelseminum is one of our best remedies. It allays promptly the irritability, and controls the excited circulation, and finally breaks up the fever entirely.

In that peculiar form of irritative fever, which is caused by some local irritation, such as ulceration, suppuration, or the presence of some foreign body, there is no remedy that will as readily control this fever (after the cause is removed) as Gelseminum, given in small doses.

In cerebro-spinal Meningitis it is especially useful in the earlier stages, given in small doses.

In measles it is one of our best remedies, controlling very much the fever and the catarrhal symptoms, at the same time determining to the surface, and also preventing spasms, which often occur.

In nervous headache, or headache from repletion, or catarrhal headache, with a full, strong pulse, Gelseminum gives very prompt relief; so, also, in neuralgic headaches.

In ophthalmia, amaurosis and sclerotitis, it acts very favorable in the inflammatory stage. In spasms of the glottis, spasmodic croup and laryngismus stridulus, we have no better remedy. In dysentery of an acute character it is a favorite remedy with me. It controls the fever, lessens the tormina and tenesmus, and thus aids in the final cure of this disease. In rigid os uteri, in cases of labor, it acts very promptly; and where the patient is threatened with convulsions while in labor, if the pulse be large, soft, and the face puffed, Gelseminum, given in doses of fifteen drops every fifteen or twenty minutes, will often prevent the spasms.

In gonorrhœa, attended with high inflammation, this is the remedy *par excellence*, and often cures the disease without the aid of any other remedies.

In such cases it may be given in ten to fifteen drops every two hours; if the discharge is thick and yellow, the canabis indica, oil of sandalwood, copabia, or cubebs, will be required. In spasmodic stricture of the urethra of a passive character, it will be found a good remedy.

Its powerfully relaxing effects point to it at once as a remedy in tetanus, either traumatic or idiopathic. In may be given in this disease in full doses—say twenty to thirty drops every two hours—until its relaxing effects are apparent. It will always cause double vision and fall of the eyelids before it dangerously affects the heart or other involuntary muscles, and should always be suspended when these effects are seen.

